# Real-world data: a brief review of the methods, applications, challenges and opportunities

**DOI:** 10.1186/s12874-022-01768-6

**Published:** 2022-11-05

**Authors:** Fang Liu, Demosthenes Panagiotakos

**Affiliations:** 1 Department of Applied and Computational Mathematics and Statistics, University of Notre Dame, 46530 Notre Dame, IN USA; 2 School of Health Sciences and Education, Harokopio University, Athens, Greece

**Keywords:** Real-world data (RWD), Real-world evidence (RWE), Electronic health records, Machine learning, Artificial intelligence, Causal inference

## Abstract

**Background:**

The increased adoption of the internet, social media, wearable devices, e-health services, and other technology-driven services in medicine and healthcare has led to the rapid generation of various types of digital data, providing a valuable data source beyond the confines of traditional clinical trials, epidemiological studies, and lab-based experiments.

**Methods:**

We provide a brief overview on the type and sources of real-world data and the common models and approaches to utilize and analyze real-world data. We discuss the challenges and opportunities of using real-world data for evidence-based decision making This review does not aim to be comprehensive or cover all aspects of the intriguing topic on RWD (from both the research and practical perspectives) but serves as a primer and provides useful sources for readers who interested in this topic.

**Results and Conclusions:**

Real-world hold great potential for generating real-world evidence for designing and conducting confirmatory trials and answering questions that may not be addressed otherwise. The voluminosity and complexity of real-world data also call for development of more appropriate, sophisticated, and innovative data processing and analysis techniques while maintaining scientific rigor in research findings, and attentions to data ethics to harness the power of real-world data.

## Introduction

Per the definition by the US FDA, real-world data (RWD) in the medical and healthcare field “are the data relating to patient health status and/or the delivery of health care routinely collected from a variety of sources”[1]. The wide usage of the internet, social media, wearable devices and mobile devices, claims and billing activities, (disease) registries, electronic health records (EHRs), product and disease registries, e-health services, and other technology-driven services, together with increased capacity in data storage, have led to the rapid generation and availability of digital RWD [2].

The increasing accessibility of RWD and the fast development of artificial intelligence (AI) and machine learning (ML) techniques, together with rising costs and recognized limitations of traditional trials, has spurred great interest in the use of RWD to enhance the efficiency of clinical research and discoveries and bridge the evidence gap between clinical research and practice. For example, during the COVID-19 pandemic, RWD are used to generate or aid the generation of real-world evidence (RWE) on the effectiveness of COVID-19 vaccination [3–5], to model localized COVID-19 control strategies [6], to characterize COVID-19 and flu using data from smartphones and wearables [7], to study behavioral and mental health changes in relation to the lockdown of public life [8], and to assist in decision and policy making, among others.

In what follows, we provide a brief review on the type and sources of RWD (Section 2) and the common models and approaches to utilize and analyze RWD (Section 3) , and discuss the challenges and opportunities of using RWD for evidence-based decision making (Section 4). This review does not aim to be comprehensive or cover all aspects of the intriguing topic on RWD (from both the research and practical perspectives) but serves as a primer and provides useful sources for readers who interested in this topic.

## Characteristics, types and applications of RWD

RWD have several characteristics as compared to data collected from randomized trials in controlled settings. First, RWD are observational as opposed to data gathered in a controlled setting. Second, many types of RWD are unstructured (e.g., texts, imaging, networks) and at times inconsistent due to entry variations across providers and health systems. Third, RWD may be generated in a high-frequency manner (e.g., measurements at the millisecond level from wearables), resulting in voluminous and dynamic data. Fourth, RWD may be incomplete and lack key endpoints for an analysis given that the original collection is not for such a purpose. For example, claims data usually do not have clinical endpoints; registry data have limited follow-ups. Fifth, RWD may be subject to bias and measurement errors (random and non-random). For example, data generated from the internet, mobile devices, and wearables can be subject to selection bias; a RWD dataset is a unrepresentative sample of the underlying population that a study intends to understand; claims data are known to contain fraudulent values. In summary, RWD are messy, incomplete, heterogeneous, and subject to different types of measurement errors and biases. A systematic scoping review of the literature suggests data quality of RWD is not consistent, and as a result quality assessments are challenging due to the complex and heterogeneous nature of these data. The sub-optimal data quality of RWD is well recognized [9–12]; how to improve it (e.g. regulatory-grade) is work in progress [13–15].

**Fig. 1:**
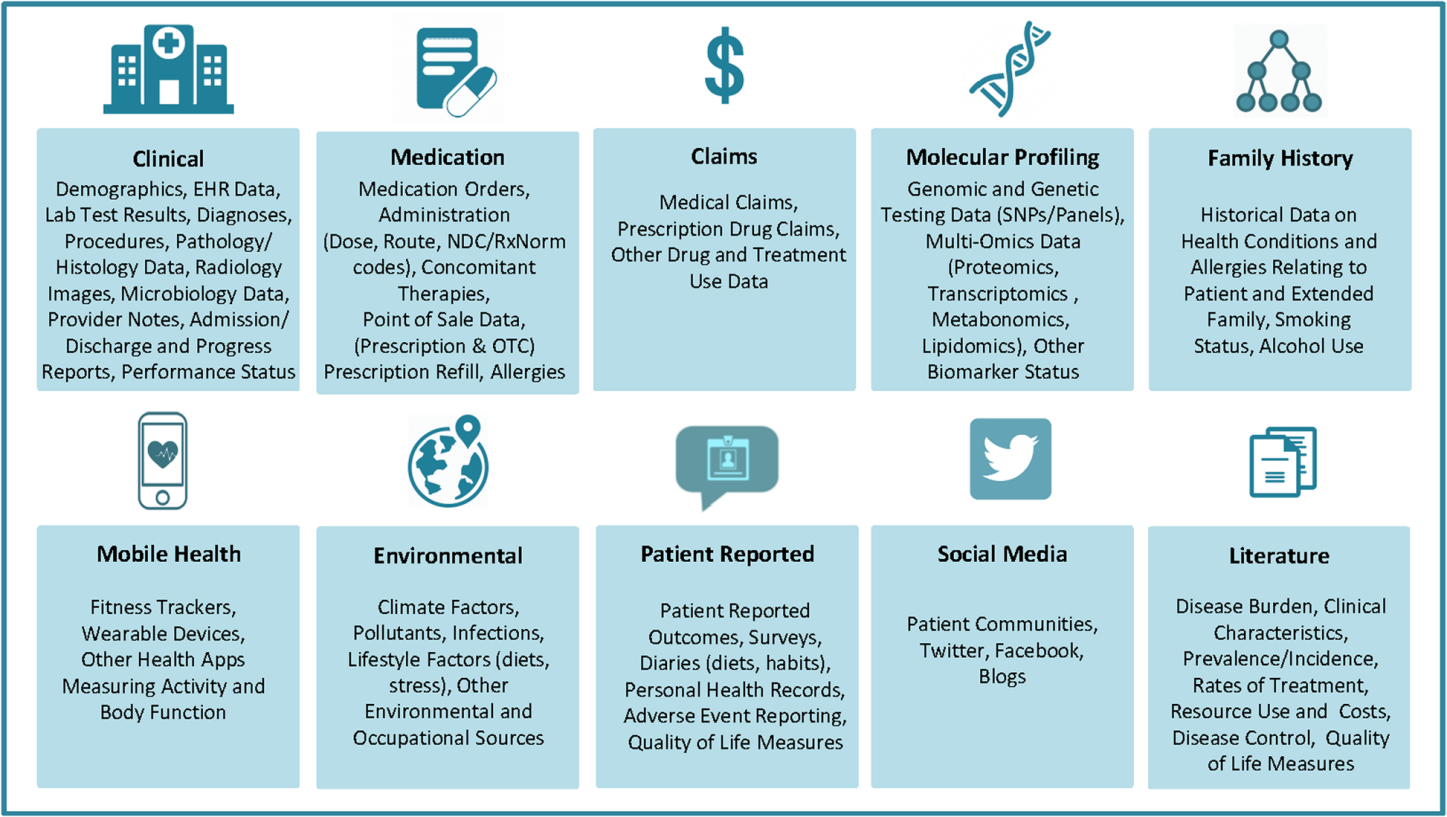
RWD Types and Sources (source: Fig. 1 in [16] with written permission by Dr. Brandon Swift to use the figure)

There are many different types of RWD. Figure 1 [16] provides a list of the RWD types and sources in medicine. We also refer readers to [11] for a comprehensive overview of the RWD data types. Here we use a few common RWD types, i.e., EHRs, registry data, claims data, patient-reported outcome (PRO) data, and data collected from wearables, as examples to demonstrate the variety of RWD and how they can be used for what purposes.

EHRs are collected as part of routine care across clinics, hospitals, and healthcare institutions. EHR data are typical RWD – noisy, heterogeneous, structured, and unstructured (e.g., text, imaging), and dynamic and require careful and intensive efforts pre-processing [17]. EHRs have created unprecedented opportunities for data-driven approaches to learn patterns, make new discoveries, assist preoperative planning, diagnostics, clinical prognostication, among others [18–27], improve predictions in selected outcomes especially if linked with administrative and claim data and usage of proper machine learning techniques [27–30], and validate and replicate findings from clinical trials [31].

Registry data have various types. For example, product registries include patients who have been exposed to a biopharmaceutical product or a medical device; health services registries consist of patients who have had a common procedure or hospitalization; and disease registries contains information about people diagnosed with a specific type of disease. Registries data enable identification and sharing best clinical practices, improve accuracy of estimates, provide valuable data for supporting regulatory decision-making [32–35]. Especially for rare diseases where clinical trials are often of small size and data are subject to high variability, registries provide a valuable data source to help understand the course of a disease, and provide critical information for confirmatory clinical trial design and translational research to develop treatments and improve patient care [34, 36, 37]. Reader may refer to [38] for a comprehensive overview on registry data and how they help understanding of patient outcomes.

Claims data refer to data generated during processing healthcare claims in health insurance plans or from practice management systems. Despite that claims data are collected and stored primarily for payment purposes originally, they have been used in healthcare to understand patients’ and prescribes’ behavior and how they interact, to estimate disease prevalence, to learn disease progression, disease diagnosis, medication usage, and drug-drug interactions, and validate and replicate findings from clinical trials [31, 39–46]. A known pitfall of claim data is fraud, on top of some of the common data characteristics of RMD, such as upcoding^1^[47]. The data fraud problem can be mitigated with detailed audits and adoption of modern statistical, data mining and ML techniques for fraud detection [48–51].

PRO data refer to data reported directly by patients on their health status. PRO data have been used to provide RWE on effectiveness of interventions, symptoms monitoring, relationships between exposure and outcomes, among others [52–55]. PRO data are subject to recall bias and large inter-individual variability.

Wearable devices generate continuous streams of data. When combined with contextual data (e.g., location data, social media), they provide an opportunity to conduct expansive research studies that are large in scale and scope [56] that would be otherwise infeasible in controlled trials. Examples of using wearable RWD to generate RWE include applications in neuroscience and environmental health [57–60]. The wearables generate huge amounts of data. Advances in data storage, real-time processing capabilities and efficient battery technology would be essential for the full utilization of wearable data.

## Using and analyzing RWD

A wide range of research methods are available to make use of RWD. In what follows, we outline a few approaches, including pragmatic clinical trials, target trial emulation, and applications of ML and AI techniques.

Pragmatic clinical trials are trials designed to test the effectiveness of an intervention in the real-world clinical setting. Pragmatic trials leverage the increasingly integrated healthcare system and may use data from EHR, claims, patient reminder systems, telephone-based care, etc. Due to the data characteristics of RWD, new guidelines and methodologies are developed to mitigate bias in RWE generated by RWD for decision making and causal inference, especially for per-protocol analysis [61, 62]. The research question under investigation in pragmatic trials is whether an intervention works in real life and trials are designed to maximize the applicability and generalizability of the intervention. Various types of outcomes can be measured in these trials, but mostly patient-centered, instead of typical measurable symptoms or markers in explanatory trials. For example, ADAPTABLE trial [63, 64] is a high-profile pragmatic trial and is the first large-scale, EHR-enabled clinical trial conducted within the U.S. It used EHR data to identify around 450,000 patients with established atherosclerotic cardiovascular disease (CVD) for recruitment and eventually enrolled about 15,000 individuals at 40 clinical centers that were randomized to two aspirin dose arms. Electronic patient follow-up for patient-reported outcomes was completed every 3 to 6 months, with a median follow-up was 26.2 months to determine the optimal dosage of aspirin in CVD patients, with the primary endpoint being the composite of all-cause mortality, hospitalization for nonfatal myocardial infarction, or hospitalization for a nonfatal stroke. The cost of ADATABLE is estimated to be only 1/5 to 1/2 of a traditional RCT of that scale.

Target trial emulation is the application of trial design and analysis principles from (target) randomized trials to the analysis of observational data [65]. By precisely specifying the target trial’s inclusion/exclusion criteria, treatment strategies, treatment assignment, causal contrast, outcomes, follow-up period, and statistical analysis, one may draw valid causal inferences about an intervention from RWD. Target trial emulation can be an important tool especially when comparative evaluation is not yet available or feasible in randomized trials. For example, [66] employs target trial emulation to evaluate real-world COVID-19 vaccine effectiveness, measured by protection against COVID-19 infection or related death, in racially and ethnically diverse, elderly populations by comparing newly vaccinated persons with matched unvaccinated controls using data from the US Department of Veterans Affairs health care system. The simulated trial was conducted with clearly defined inclusion/exclusion criteria, identification of matched controls, including matching based on propensity scores with careful selection of model covariates. Target trial emulation has also been used to evaluate the effect of colon cancer screening on cancer incidence over eight years of follow up [67], and the risk of urinary tract infection among diabetic patients [68].

RWD can also be used as historical controls and reference groups for controlled trials, with assessment of the quality and appropriateness of the RWD and employment of proper statistical approaches for analyzing the data [69]. Controlling for selection bias and confounding is key to the validity of this approach because of the lack of randomization and potentially unrecognized baseline differences, and the control group needs to be comparable with the treated group. RWD also provide a great opportunity to study rare events given the data voluminousness [70–72]. These studies also highlight the need for improving the RWD data quality, developing surrogate endpoints, and standardizing data collection for outcome measures in registries.

In terms of analysis of RWD, statistical models and inferential approaches are necessary for making sense of RWD, obtaining causal relationships, testing/validating hypotheses, and generating regulatory-grade RWE to inform policymakers and regulators in decision making – just as in the controlled trial settings. In fact, the motivation for and the design and analysis principles in pragmatic trials and target trial emulation are to obtain causal inference, with more innovative methods beyond the traditional statistical methods to adjust for potential confounders and improve the capabilities of RWD for causal inference [73–76].

ML techniques are getting increasingly popular and are powerful tools for predictive modeling. One reason for their popularity is that the modern ML techniques are very capable of dealing with voluminous, messy, multi-modal, and various unstructured data types without strong assumptions about the distribution of data. For example, deep learning can learn abstract representations of large, complex, and unstructured data; natural language processing (NLP) and embedding methods can be used to process texts and clinical notes in EHRs and transform them to real-valued vectors for downstream learning tasks. Secondly, new and more powerful ML techniques are being developed rapidly, due to the high demand and the large group of researchers in the field attracted by the hot topic. Thirdly, there are also many open source codes (e.g., on Github) and software libraries (e.g., TensorFlow, Pytorch, Keras) out there to facilitate the implementation of these techniques. Indeed, ML has enjoyed a rapid surge in the last decade or so for a wide range of applications in RWD, outperforming more conventional approaches [77–85]. For example, ML is widely applied in in health informatics to generate RWE and formulate personalized healthcare [86–90] and was successfully employed on RWD collected during the COVID-19 pandemic to help understand the disease and evaluate its prevention and treatment strategies [91–95]. It should be noted that the ML techniques are largely used for predictions and classification (e.g., disease diagnosis), variable selections (e.g, biomarker screening), data visualization, etc, rather than generating regulatory-level RWE; but this may change soon as regulatory agencies are aggressively evaluating ML/AI for generating RWE and engaging stakeholders on the topic [96–99].

It would be more effective and powerful to combine the expertise from statistical inference and ML when it comes to generating RWE and learning causal relationships. One of the recent methodological developments is indeed in that direction – leveraging the advances in semi-parametric and empirical process theory and incorporating the benefits of ML into comparative effectiveness using RWD. A well-known framework is targeted learning [100–102] that has been successfully applied in causal inference for dynamic treatment rules using EHR data [103] and efficacy of COVID-19 treatments [104], among others.

Regardless of which area a RWD project focuses on – causal inference or prediction and classification, representativeness of RWD of the population where the conclusions from the RWD project will be generalized to is critical. Otherwise, estimation or prediction can be misleading or even harmful. The information in RWD might not be adequate to validate the appropriateness of the data for generalization; in that case, the investigators should resist the temptation to generalize to groups that they are unsure about.

## Challenges and opportunities

Various challenges – from data gathering to data quality control to decision making – still exist in all stages of a RWD life cycle despite all the excitement around their transformative potentials. We list some of the challenges below, where plenty of opportunities for improvement exist and greater efforts are needed to harness the power of RWD.

*Data quality*: RWD are now often used for other purposes than what they are originally collected for and thus may lack information for critical endpoints and not always be positioned for generating regulatory-grade evidence. On top of that, RWD are messy, heterogeneous, and subject to various measurement errors, all of which contribute to the lower quality of RWD compared to data from controlled trials. As a result, accuracy and precision of results based on RWD are negatively impacted and misleading results or false conclusions can be generated. While these do not preclude the use of RWD in evidence generation and decision making, data quality issues need to be consistently documented and addressed as much as possible through data cleaning and pre-processing (e.g., imputation to fill in missing values, over-sampling for imbalanced data, denoising, combining disparate pieces of information across databases, etc). If an issue can be addressed during the pre-processing stage, efforts should be made to correct it during data analysis or caution should be used when interpreting the results. Early engagement of key stakeholders (e.g., regulatory agencies if needed, research institutes, industries etc.) are encouraged to establish data quality standards and reduce unforeseen risks and issues.

*Efficient and practical ML and statistical procedures*: Fast growth of digital medical data and the fact that workforce and investment flood into the field also drive the rapid development and adoption of modern statistical procedures and ML algorithms to analyze the data. The availability of open-source platforms and software greatly facilitate the application of the procedures in practice. On the other hand, noisiness, heterogeneity, incompleteness, and unbalancedness of RWD may cause considerable under-performance of the existing statistical and ML procedures and demand new procedures that target specifically at RWD and can be effectively deployed in the real world. Further, the availability of the open-source platform and software and the accompanied convenience, while offered with good intentions, also increases the chance of practitioners misusing the procedures, if not equipped with proper training or understanding the principles of the techniques before applying them to real-world situations. In addition, to maintain scientific rigor during the RWE generation process from RWD, results from statistical and ML procedures would require medical validation either using expert knowledge or conducting reproducibility and replicability studies before they are being used for decision making in the real world [105].

*Explainability and interpretability*: Modern ML approaches are often employed in a black-box fashion and there a lack of understanding of the relationships between input and output and causal effects. Model selection, parameter initialization, and hyper-parameter tuning are also often conducted in a trial-and-error manner, without domain expert input. This is in contrast to the medical and healthcare field where interpretability is critical to building patient/user trust, and doctors are unlikely to use technology that they don’t understand. Promising and encouraging research work on this topic has already started [106–111], but more research is warranted.

*Reproducibility and replicability*: Reproducibility and replicability^2^ are major principles in scientific research, RWD included. If an analytical procedure is not robust and its output is not reproducible or replicable, the public would call into questions the scientific rigor of the work and doubt the conclusion from a RWD-based study [113–115]. Result validation, reproducibility, and replicability can be challenging given their messiness, incompleteness, unstructured data, but need to be established especially considering that the generated evidence could be used towards regulatory decisions and affect the lives of millions of people. Irreproducibility can be mitigated by sharing raw and processed data and codes, assuming no privacy is compromised in this process. For replicability, given that RWD are not generated from controlled trials and every data set may has its own unique data characteristics, complete replicability can be difficult or even infeasible. Nevertheless, detailed documentation of data characteristics and pre-processing, pre-registration of analysis procedures, and adherence to open science principles (e.g., code repositories [116]) are critical for replicating findings on different RWD datasets, assuming they come from the same underlying population. Readers may refer to [117–119] for more suggestions and discussions on this topic.

*Privacy*: Ethical issues exist when an RWD project is implemented, among which, privacy is a commonly discussed topic. Information in RWD is often sensitive, such as medical histories, disease status, financial situations, and social behaviors, among others. Privacy risk can increase dramatically when different databases (e.g., EHR, wearables, claims) are linked together, a common practice in the analysis of RWD. Data users and policymakers should make every effort to ensure that RWD collection, storage, sharing, and analysis follow established data privacy principles (i.e., lawfulness, fairness, purpose limitation, and data minimization). In addition, privacy-enhancing technology and privacy-preserving data sharing and analysis can be deployed, where there already exist plenty effective and well-accepted state-of-the-art concepts and approaches, such as differential privacy^3^[120] and federated learning^4^[121, 122]. Investigators and policymakers may consider integrating these concepts and technology when collecting and analyzing RWD and disseminating the results and RWE from the RWD.

*Diversity, Equity, Algorithmic fairness, and Transparency (DEAT)*: DEAT is another important ethical issue to consider in an RWD project. RWD may contain information from various demographic groups, which can be used to generate RWE with improved generalizability compared to data collected in controlled settings. On the other hand, certain types of RWD may be heavily biased and unbalanced toward a certain group, not as diverse or inclusive, and in some cases, even exacerbate disparity (e.g., wearables and access to facilities and treatment may be limited to certain demographic groups). Greater effort will be needed to gain access to RWD from underrepresented groups and to effectively take into account the heterogeneity in RWD while being mindful of the limitation for diversity/equity. This topic also relates to algorithmic fairness, which aims at understanding and preventing bias in ML models. Algorithmic fairness is an increasingly popular research topic in literature [123–127]. Incorrect and misleading conclusions may be drawn if the trained models systematically disadvantage a certain group (e.g., a trained algorithm might be less likely to detect cancer in black patients than white patients or in men than women). Transparency means that information and communication concerning the processing of personal data must be easily accessible and easy to understand. Transparency ensures that data contributors are aware of how their data are being used and for what purposes and decision-makers can evaluate the quality of the methods and the applicability of the generated RWE [128–131]. Being transparent when working with RWD is critical for building trust among the key stakeholders during an RWD life cycle (individuals who supply the data, those who collect and manage the data, data curators who design studies and analyze the data, and decision and policy makers).

**Fig. 2:**
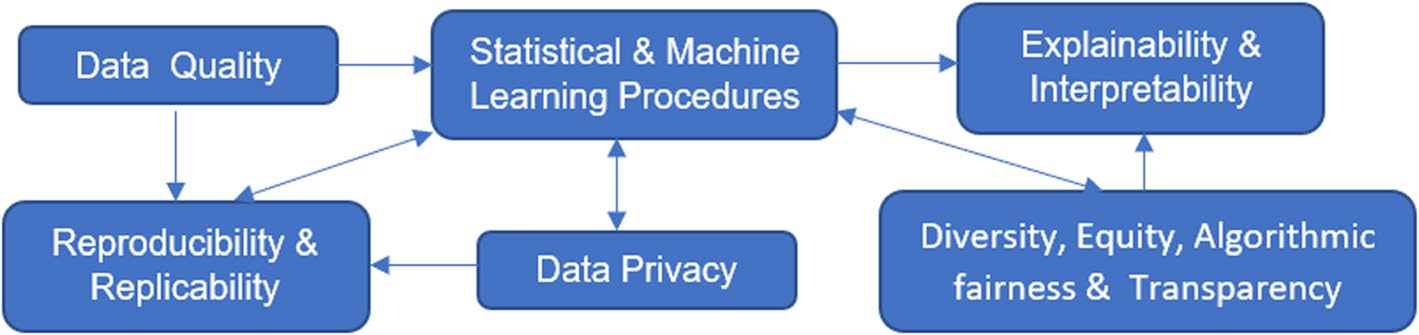
Challenges in RWD and Their Relations

The above challenges are not isolated but rather connected as depicted in Fig. 2. Data quality affects the performance of statistical and ML procedures; data sources and the cleaning and pre-processing process relate to result reproducibility and replicability. How data are analyzed and which statistical and ML procedures to use have an impact on reproducibility and replicability, whether privacy-preserving procedures are used during data collected and analysis and how information is shared and released relate to data privacy, DEAT, and explainability and interpretability, which can in turns affect which ML procedures to apply and development of new ML techniques.

## Conclusions

RWD provide a valuable and rich data source beyond the confines of traditional epidemiological studies, clinical trials, and lab-based experiments, with lower cost in data collection compared to the latter. If used and analyzed appropriately, RWD have the potential to generate valid and unbiased RWE with savings in both cost and time, compared to controlled trials, and to enhance the efficiency of medical and health-related research and decision-making. Procedures that improve the quality of the data and overcome the limitation of RWD to make the best of them have been and will continue to be developed. With the enthusiasm, commitment, and investment in RWD from all key stakeholders, we hope that the day that RWD unleashes its full potential will come soon.

## Data Availability

Not applicable. This is a review article. No data or materials were generated or collected.

## Abbreviations

AI: artificial intelligence
CVD: cardiovascular disease
COVID: coronavirus disease
DEAT: diversity, equity, algorithmic fairness, and transparency
EHR: electronic health records
ML: machine learning
NLP: natural language processing
PRO: patient-reported outcome
RWD: real-world data
RWE: real-world evidence

## Footnotes

1 Upcoding refers to instances in which a medical service provider obtains additional reimbursement from insurance by coding a service it provided as a more expensive service than what was actually performed

2 Reproducibility refers to “instances in which the original researcher’s data and computer codes are used to regenerate the results” and replicability refers to “instances in which a researcher collects new data to arrive at the same scientific findings as a previous study.” [112]

3 Differential privacy provides a mathematically rigorous framework in which randomized procedures are used to guarantee individual privacy when releasing information.

4 Federated learning enables local devices to collaboratively learn a shared model while keeping all training data on the local devices without sharing, mitigating privacy risks.
