# Correction: Real‑world data: a brief review of the methods, applications, challenges and opportunities

**DOI:** 10.1186/s12874-023-01937-1

**Published:** 2023-05-02

**Authors:** Fang Liu, Demosthenes Panagiotakos

**Affiliations:** 1 Department of Applied and Computational Mathematics and Statistics, University of Notre Dame, Notre Dame, IN 46530 USA; 2 School of Health Sciences and Education, Harokopio University, Athens, Greece

**Correction: BMC Medical Research Methodology 22, 287 (2022)**

10.1186/s12874-022-01768-6

Following publication of the original article [1], the authors identified an error in the author name of Demosthenes Panagiotakos. The given name and family name were erroneously transposed.

The incorrect author name is: Panagiotakos Demosthenes

The correct author name is: Demosthenes Panagiotakos

The author group has been updated above and the original article [1] has been corrected.

## References

[1] Liu F, Panagiotakos D (2022). Real-world data: a brief review of the methods, applications, challenges and opportunities. BMC Med Res Methodol.

